# Germline Transgenic Pigs by *Sleeping Beauty* Transposition in Porcine Zygotes and Targeted Integration in the Pig Genome

**DOI:** 10.1371/journal.pone.0023573

**Published:** 2011-08-29

**Authors:** Wiebke Garrels, Lajos Mátés, Stephanie Holler, Anna Dalda, Ulrike Taylor, Björn Petersen, Heiner Niemann, Zsuzsanna Izsvák, Zoltán Ivics, Wilfried A. Kues

**Affiliations:** 1 Institut für Nutztiergenetik, Friedrich-Loeffler-Institut, Neustadt, Germany; 2 Max Delbrück Center for Molecular Medicine, Berlin, Germany; 3 University of Debrecen, Debrecen, Hungary; Wellcome Trust Centre for Stem Cell Research, United Kingdom

## Abstract

Genetic engineering can expand the utility of pigs for modeling human diseases, and for developing advanced therapeutic approaches. However, the inefficient production of transgenic pigs represents a technological bottleneck. Here, we assessed the hyperactive *Sleeping Beauty* (*SB100X*) transposon system for enzyme-catalyzed transgene integration into the embryonic porcine genome. The components of the transposon vector system were microinjected as circular plasmids into the cytoplasm of porcine zygotes, resulting in high frequencies of transgenic fetuses and piglets. The transgenic animals showed normal development and persistent reporter gene expression for >12 months. Molecular hallmarks of transposition were confirmed by analysis of 25 genomic insertion sites. We demonstrate germ-line transmission, segregation of individual transposons, and continued, copy number-dependent transgene expression in F1-offspring. In addition, we demonstrate target-selected gene insertion into transposon-tagged genomic loci by Cre-*loxP*-based cassette exchange in somatic cells followed by nuclear transfer. Transposase-catalyzed transgenesis in a large mammalian species expands the arsenal of transgenic technologies for use in domestic animals and will facilitate the development of large animal models for human diseases.

## Introduction

The pig is an important model in biomedical research [1]–[5]. Pigs have been used as models for cardiovascular disease [6], atherosclerosis [7], wound repair [8], cancer [9], diabetes [10] and ophthalmological diseases [1]. Porcine physiology, metabolism, genome organisation, life span and pathology reflect human pathophysiology much better than small animal models. The prospect of the pig as a large animal model is further underscored by the recent completion of a raw draft of the porcine genome (www.sanger.ac.uk/Projects/S_scrofa). Genetic engineering can expand the utility of pigs for modeling human diseases [1], [4], for developing and validating novel clinical treatments, or for providing tissue for xenotransplantation [11]. This requires translation of the repertoire of genetic tools currently employed in smaller model organisms to practical use in domestic pigs.

Transgenesis in the pig, most commonly achieved by pronuclear DNA injection or, alternatively, by somatic cell nuclear transfer (SCNT), is an inefficient and expensive process hampered by poor predictability of the patterns and levels of transgene expression [2], [12], [13]. After pronuclear DNA-injection into porcine zygotes, typically only 1% of the treated embryos develop into transgenic founders [12]. Similarly, SCNT in pigs is encumbered by low developmental potential of reconstructed embryos, and no more than 1–3% of reconstructed embryos develop to term [4], [14]–[19]. Only a fraction of gain-of-function transgenic offspring produced by these methods shows the desired expression patterns of the transgene, as integration of transgenes occurs randomly in the genome [20], [21]. As a consequence, the transgene may be silenced by epigenetic mechanisms or may show a variegated expression pattern in a non-predictable fashion. Currently, detailed screening of founder animals is the only way to identify animals with suitable expression patterns. The long generation interval of pigs (nearly one year) and the high costs of animal housing coupled with the low efficiency of transgenesis limit a broader application of porcine genetic engineering.

Class II DNA transposons have been successfully used for transgenesis and insertional mutagenesis in several invertebrate models [22]. The discovery of the *Sleeping Beauty* (*SB*) transposon [23] expanded the utility of transposon-based technologies in vertebrate species. During transposition, a single copy of a gene of interest flanked by the inverted terminal repeats (ITR) of a transposon is stably incorporated into the genome by the enzymatic factor (transposase) of transposition. The drawbacks of classical methods for transgenesis can be overcome by utilizing transposase-catalyzed gene delivery, as it increases the efficiency of chromosomal integration and preferentially favours single-copy (monomeric) insertion events. Commonly, *in vitro* synthesized transposase mRNA is injected together with a plasmid-based transposon, in some cases both components are delivered as DNA. Germline transgenesis has been achieved in invertebratae, fish, frogs, chicken and mammals employing the transposons *Minos*, *Tol1*, *Tol2*, *piggyBac* and *Sleeping Beauty* [24]–[32]. Pronuclear injections of a hyperactive version of the *SB* transposase (*SB100X*) were shown to be highly efficient in mice [33]. Recently, porcine primary cells were transduced with SB plasmids, encoding antibiotic selection markers, and antibiotic-resistant cells were successfully employed to generate cloned pigs by SCNT [34], [35].

Here, we show that cytoplasmic injection of zygotes with plasmids encoding both components of the hyperactive *SB100X* system is a simple and highly efficient method for porcine transgenesis without the necessity of an antibiotic selection marker. This transposon-based technique represents a direct and efficient route to germline-competent founders to establish transgenic lines in large animal models.

## Results

**Figure 1:**
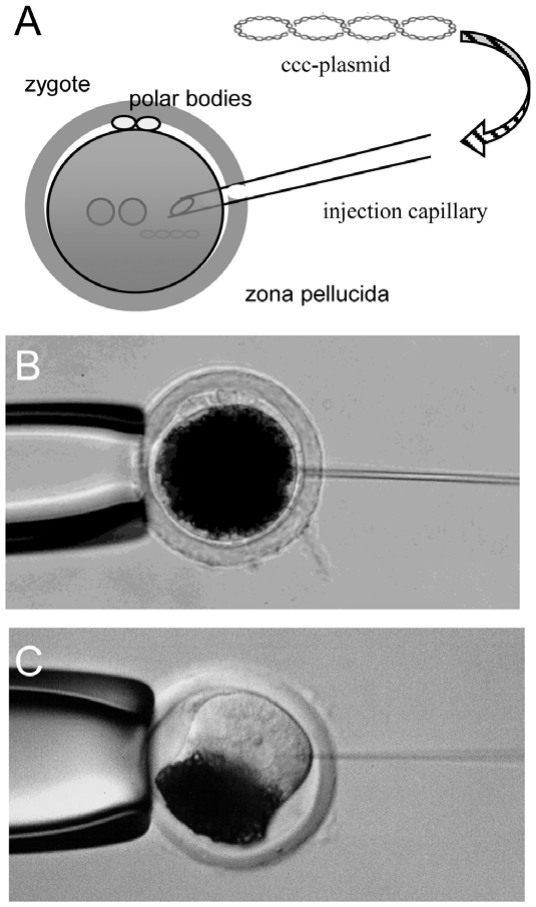
Injection of ccc-plasmids into the cytoplasm of a zygote. A) Schematic depiction of cytoplasmic plasmid injection (CPI) into an opaque zygote. B) Cytoplasmic injection into a porcine zygote. C) For comparative reasons a pronuclear injection in a porcine zygote is shown. To reveal the pronuclei, a high speed centrifugation at 12'000–15'000 g is necessary.

We explored cytoplasmic injection of covalently closed circular (ccc)-plasmid constructs [36] to deliver the two components of the hyperactive *Sleeping Beauty* (*SB100X*) transposon system into zygotes for the generation of transgenic pigs. A plasmid expressing the *SB100X* transposase (pCMV-SB100X) and a transposon donor plasmid carrying a *Venus* fluorophore driven by the ubiquitous CAGGS promoter and flanked by heterospecific *loxP* sites (pT2/VenusRMCE, Figure S1) were co-injected into the cytoplasm of porcine zygotes (Fig. 1). Intact zygotes were transferred into the oviduct of recipient animals, and fetuses from day 30 of gestation (d30) as well as founders born at term were analysed for their transgenic status and *Venus* expression. In addition, germline transmission of the transposon transgene was assessed.

In all experiments approximately 10 picoliter (pl) of a plasmid solution (see Table 1 for concentrations) was microinjected directly into the cytoplasm. Cytoplasmic injection has the advantage that high-speed centrifugation at 12000–15000 g [12] is not required to reveal the pronuclei within the opaque porcine zygotes (Fig. 1). In some cases, zygotes and unintendendly 2-cell embryos were flushed from the oviduct, then the DNA was injected into one blastomere of 2-cell stage embryos. Previous own studies suggested that transfer of 30–40 microinjected embryos is optimal for the establishment of a pregnancy in the pig [21]. Thus on days where zygotes and 2-cell stages were flushed, both stages were injected and groups of optimal size were pooled and transferred.Three different groups of injection mixtures were tested (Table 1). In groups A and B different ratios of pT2/VenusRMCE and pCMV-SB100X were injected. In group C, a mixture of pT2/VenusRMCE plasmid and synthetic *SB100X* mRNA was injected, since injection solutions of this composition had been used successfully before for pronuclear injections in murine zygotes [33].

**Table 1:** Injection parameters and rates of transgenesis.

	Injection solution	experim-ental day	no. of flushed embryos	no. of injected embryos	no. of transferred embryos	no. of recipients/pregnant recipients	no. of recovered fetuses (day 30) or born piglets at term	no of transgenic fetuses or piglets	ratio of transgenic animals or fetuses per offspring/per injected zygote (%)
**A**	pT2/VenusRMCE (10 fg/pl)	1	30 zygotes	47	43	2/2	6 fetuses	4 fetuses	57.1/
	pCMV-SB100X (5 fg/pl)		18×2-cells				+1 “degenerated”	+2 mosaic transg	8.5
								(amnion)	
		2	72 zygotes	86	77	2/1	12 piglets	5 piglets	41.7/
			14×2-cells				(2 stillborn)	(1 stillborn)	
			27 oocytes						
	**subtotal A**			**133**	**120**	**4/3**	**19**	**9**	**47.3/**
									**6.8**
**B**	pT2/VenusRMCE (10 fg/pl)	3	30 zygotes	30	21	1/0	n.a.	n.a.	n.a.
	pCMV-SB100X (2.5 fg/pl)		9×2-cells						
		4	49 zygotes	49	44	1/1	5 fetuses	0	0
	**subtotal B**			**79**	**65**	**2/1**	**5**	**0**	**0**
**C**	pT2/VenusRMCE (2 fg/pl)	5	50 zygotes	58	57	2/1	17 fetuses	0	0
	SB100X-mRNA (5 fg/pl)		10×2-cells						

n.a., not applicable.

Treatment A resulted in 4 transgenic fetuses out of 7, and 5 transgenic piglets out of 12 born (Table 1). In groups B and C no transgenic fetuses could be recovered, most likely due to reduced plasmid concentrations and a higher susceptibility of the synthetic RNA to enzymatic degradation. The transgenic fetuses and piglets resulting from group A were investigated for phenotype, genotype and germline transmission.

**Figure 2:**
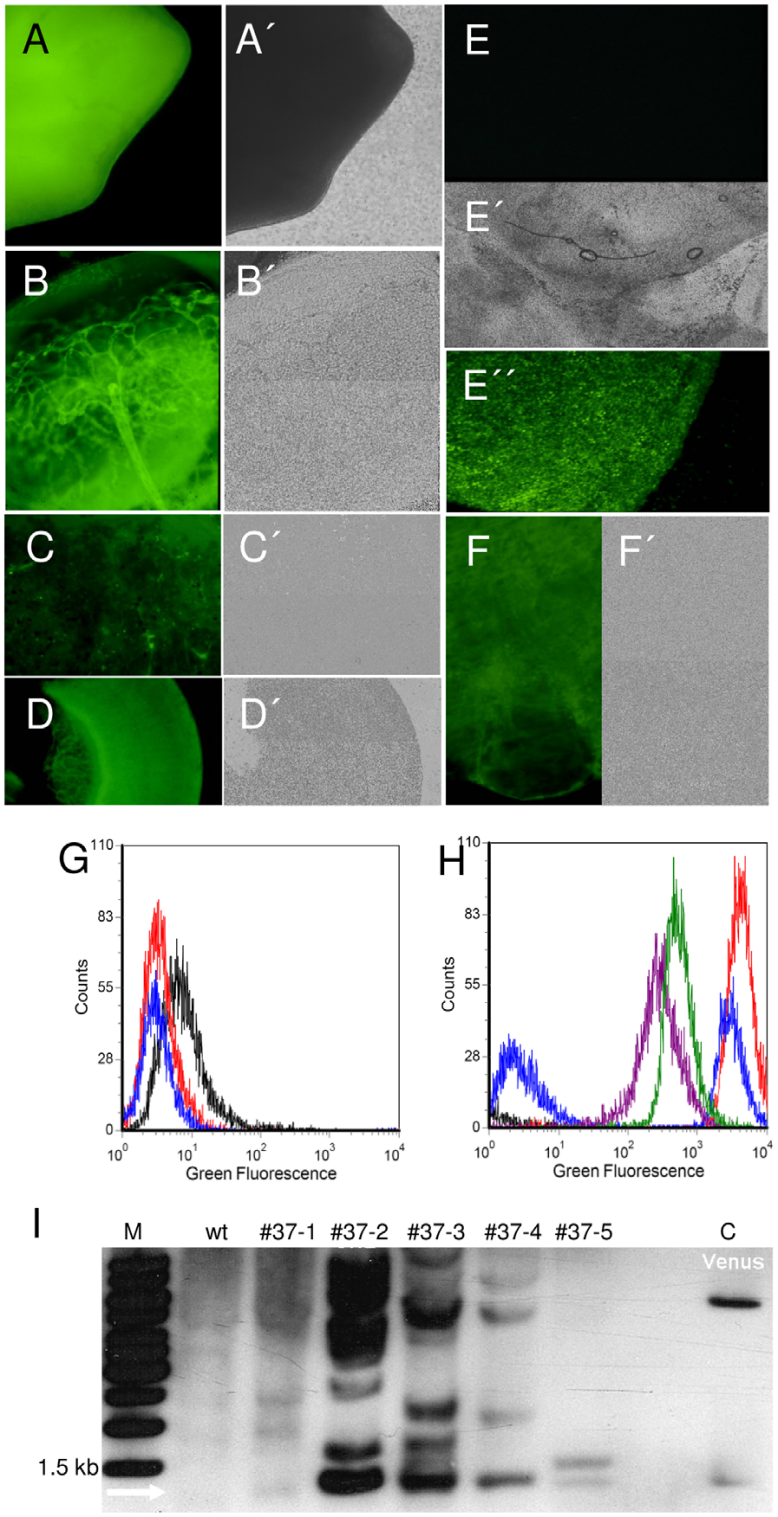
*Venus*–transposon expression in tissues of the three germ layers and extraembryonic membranes. Images of selected organs of F0 transgenic porcine fetuses (d30) derived from cytoplasmic injection of transposon plasmids into zygotes. **A–F**, specific excitation of the *Venus* fluorophore; **A′–F′**, corresponding bright field images. A, limb bud; B, eye; C, heart; D, intestine; E, E′, amnion of a non-transgenic fetus, E″, amnion of a transgenic fetus; F, mesonephros. **G**) Flow cytometric determination of *Venus*-fluorescence. Black line represents wildtype fibroblasts, blue and red lines, non-transgenic fibroblasts from fetuses #37-1 and #40-1. **H**) Flow cytometry of *Venus*-transposon transgenic fibroblasts. Red, blue, green and purple lines, fibroblasts from fetuses #37-2, #37-3, #37-4 and #37-5, respectively. The difference of fluorescence intensities between wildtype (G) and transgenic fibroblasts was so great that the transgenic fibroblasts were measured with reduced gain settings. Fibroblasts of #37-3 (blue) were mosaic, approximately 50% of the cells displayed a reduced fluorescence. I) Southern blot of fetal fibroblasts. M, molecular size marker; wt, wild type DNA; #37-1 to #37-5, fetal IDs; c, positive control: Venus plasmid digested with NcoI. The NcoI digest produces a constant fragment of 1.4 kb and a variable fragment >1.4 kb.

**Figure 3:**
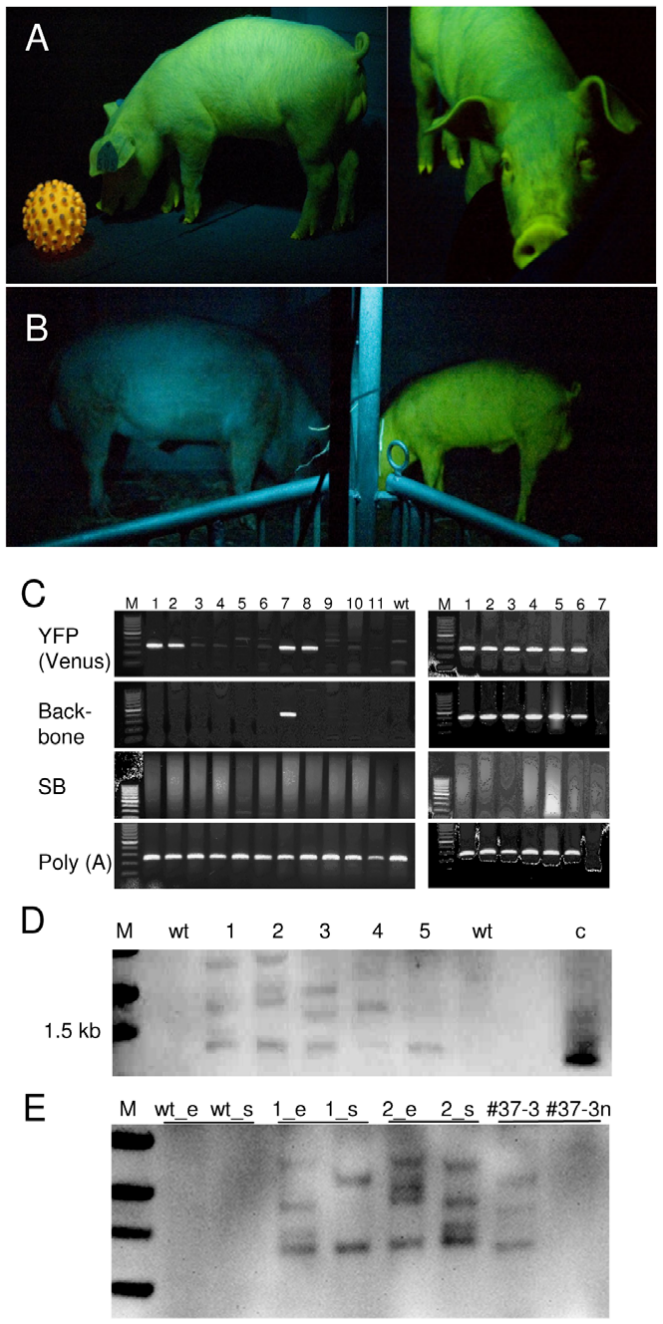
Persistent transgene expression in transposon-transgenic pigs. **A**) Transgenic boar (#505) viewed under specific excitation from side and front at the age of 2 months playing with an auto-fluorescent toy ball (left). **B**) Wildtype boar (left) and transgenic boar (#503, right, 8 months of age) photographed side-by-side under a light source with specific excitation of the *Venus* fluorophore. The animals are separated by a fence visible in the middle of the image. Blue appearance of wildtype animal is due to reflected and scattered excitation light. **C**) PCR genotyping of ear biopsies of born piglets for the presence of *Venus*, plasmid backbone; *SB100X* transposase, and control amplicons [poly(A) polymerase, POL(A)]. M, size marker, lane 1 (#505), lane 2 (#503), lanes 3–11 littermates (no 3-11); wt, wildtype pig sample. Lane 7 and lane 8 correspond to deceased piglets with *Venus*-fluorescence. Right, genotyping of different organs from stillborn piglet (no 12) with *Venus* fluorescence: M, size marker; 1, ear; 2, heart, 3, muscle; 4, spleen; 5, kidney; 6, liver, 7, no template. **D**) Southern blot of transgenic piglets. Genomic DNA was isolated from ear biopsies, NcoI digested and blotted with the Venus probe. M, molecular size marker; wt, wildtype; 1–5, genomic DNA from transgenic piglets; c, Venus plasmid control. **E**) Analysis of cell chimerism; wild type (wt_e and wt_s) and transgenic boars (#503: 1_e and 1_s; #505: 2_e and 2_s) genomic DNA from ear biopsies (_e) and spermatozoa (_s) was blotted and hybridized with the Venus probe. Note the different fragment patterns between tissues of the founders. In addition genomic DNA from total fibroblasts of fetus #37-5 (#37-5) and of the cell fraction sorted for absence of Venus fluorescence was probed (#37-5n).

Phenotypic analysis of the transgenic fetuses by specific excitation of the *Venus* fluorophore revealed that almost all cell types expressed the transgene (Fig. 2). Four fetuses showed *Venus* fluorescence in ecto-, endo- and mesodermal organs and extraembryonal membranes. One fetus showed visible *Venus*-fluorescence only in the amnion, but not in the fetus itself. Flow cytometric analysis of fetal fibroblast cultures revealed distinct populations of *Venus*-fluorescent cells (Fig. 2H). One cell culture (#37-3) appeared to be mosaic, with a highly positive and a negative cell population (Fig. 2H). Flow cytometric sorting and PCR genotyping suggested that the Venus-negative population was non-transgenic. This was confirmed by Southern blotting (see below, Fig. 3E).

Molecular analysis by Southern blotting and PCR confirmed that phenotypically positive fetuses carried the *Venus*-transposon, but not transposon plasmid backbone sequences (Table S1), indicating a transposase-dependent integration mechanism. Southern blotting with *SB100X* probes did not result in specific hybridization signals (Table S1), indicating absence of these sequences in the fetuses. Only one amnion sample was found to be positive for the pCMV-SB100X amplicon, suggesting a passive, non-transposase-mediated integration. Southern blotting with a *Venus*-specific probe revealed one integration in fetus #37-5, three integrations in #37-4, ∼five integrations in #37-3 and >10 integrations in fetus #37-2 (Fig. 2I). The copy number of the integrated transposons correlated with the intensities of *Venus* fluorescence as determined by FACS analysis (fluorescence intensities: #37-2>#37-3>#37-4>#37-5) (Fig. 2H). Cloning and sequencing of 25 integration sites from fetuses, founders and their offspring (see below) by splinkerette PCR confirmed specific *SB*-catalyzed transposition events at the expected TA target dinucleotide sites (Table S2). Due to the relatively short genomic sequences flanking the integrated transposons and the existing gaps in the porcine genome sequence, only five out of the 25 identified integration sites could be assigned to their chromosomal positions; these mapped to porcine chromosomes X, 3, 7, 8, and 13 (Table S2). Four of these were found in intergenic regions of chromosomes X (Figure S2), 3, 7 and 13, and one integration site was located within intron 2 of SMARCA5 (SWI/SNF related, matrix associated actin dependent regulator of chromatin) gene on chromosome 8 (Table S2; Figure S2).

Embryo transfer of group A zygotes resulted in the birth of 12 piglets at full term, of which two were still born. All piglets were normally developed, and did not show any abnormalities. Phenotypically, four of the vital piglets and one of the stillborns showed strong and homogenous *Venus*-fluorescence in all surface areas (Figs. 3A and 3B). Due to a bacterial infection two transgenic and three non-transgenic animals died shortly after birth. *Venus*-fluorescence of skin, tongue, claws and eyes of two vital male pigs (unique ear tag numbers #503 and #505) did not change over a period of >12 months, and did not compromise growth, behaviour or reproductive parameters. PCR genotyping confirmed that all phenotypically positive animals carried the *Venus*-transposon, but were negative for *SB100X*–sequences (Fig. 3C). Two of the piglets (lane 7 in Fig. 3C and right panel) were also positive for backbone sequences of the *Venus*-transposon-plasmid. However, the other animals did not carry backbone sequences (Figure S3). Monomeric copy numbers of 3, 2 and 1 were found in ear tag biopsies of the transgenic piglets, respectively (Fig. 3 D). A molecular analysis of the founder boars #503 and #505, however, suggested that albeit the analyzed tissue biopsies (ear biopsy and sperm) are transgenic, the integration sites seem to differ between tissues (Fig. 3E).

**Figure 4:**
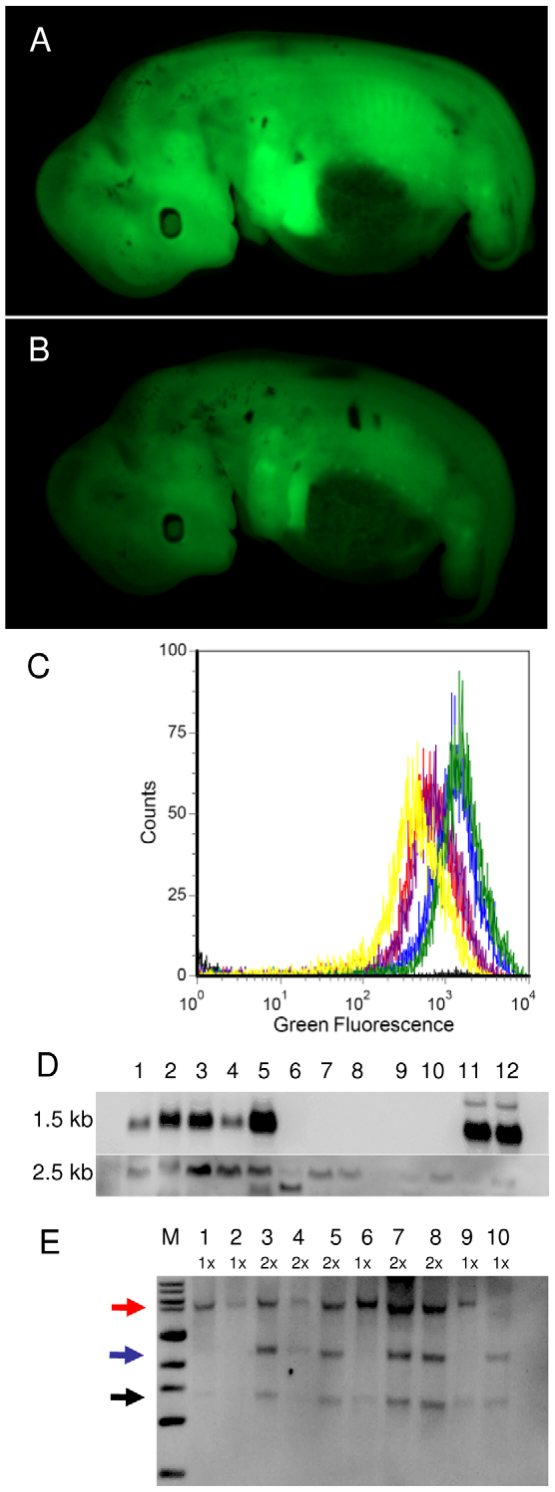
Segregation of *Venus*-transposons in F1-fetuses. Fetuses derived from insemination of a wildtype sow with semen of transgenic boar #503 were isolated at day 29 p.c., and fluorescence images were taken under normalized conditions. **A**) Typical image of a strongly fluorescent fetus (F1-2), **B**) Typical image of a weakly fluorescent fetus (F1-4). The fluorescence intensities correlated with the transposon copy numbers, as determined by Southern blotting. **C**) Flow cytometric measurements of *Venus*-fluorescence in fibroblasts derived from F1-fetuses with weak fluorescence intensity: F1-1 (red), F1-5 (purple), F1-11 (yellow) and strongly fluorescent fetuses: F1-5 (blue) and F1-9 (green). **D**) Expression of *Venus* in different tissues of d29 porcine fetus (F1-3, strongly fluorescent) as determined by Northern blotting with a *Venus*-specific probe (top): head (1); carcasse (2); mesonephros (3); liver (4); heart/lung (5) and control samples from wildtype pig: heart (6); lung (7); liver (8). In addition, RNAs from wildtype murine heart (9) and lung (10); and RNAs from *Venus*-transposon transgenic murine heart (11) and lung (12) were loaded. Bottom, reprobed blot with an actin-specific probe. Porcine tissues show organ-specific splice patterns of actin transcripts [51]. **E**) Segregation of *Venus*-transposons in F1-animals. Genomic DNA from F1-fetuses was analysed by Southern blot with the *Venus*-probe. M, size marker; 1–10, genomic DNA from ten F1 offspring. Black arrow, internal, constant band at ∼1.4 kb; blue arrow, external fragment of one integrant; red arrow, external fragment of the other integrant. 1× and 2× indicate the deduced transposon copy numbers.

To test germline transmission potential of transgenic founders, wildtype sows were inseminated with semen from boar #503, and a total of 18 normally developed F1-fetuses were recovered from three sacrificed sows. Sixteen F1-fetuses were *Venus*-positive and showed a clear grading of fluorescence intensities (Figs. 4A and 4B), whereas two fetuses were fluorescence-negative. Flow cytometric analysis of fetal cell cultures derived from 5 of the positive F1-fetuses revealed that their fluorescence intensities split into two separate classes (Fig. 4C). The more strongly fluorescent fibroblasts had approximately double the intensity of fluorescence compared to the weaker ones. Southern blotting suggested that the weakly fluorescent fetuses carried one *Venus*-transposon copy, whereas the strongly fluorescent fetuses carried two transposon copies (Fig. 4E). Ubiquitous expression of *Venus*-transcripts was shown by Northern blotting (Fig. 4D), albeit some variability of transcript levels can be detected in different organs. One F1 litter was delivered at term and 8 healthy piglets were born, of which 6 were transgenic and *Venus*-expressing (Table S1 and Video S1). Together the data indicate (i) *SB*-catalyzed integration, (ii) germline transmission of chromosomally integrated transposons, (iii) segregation of the transposon integrations and (iv) copy number dependent fluorescence in F1 offspring.

**Figure 5:**
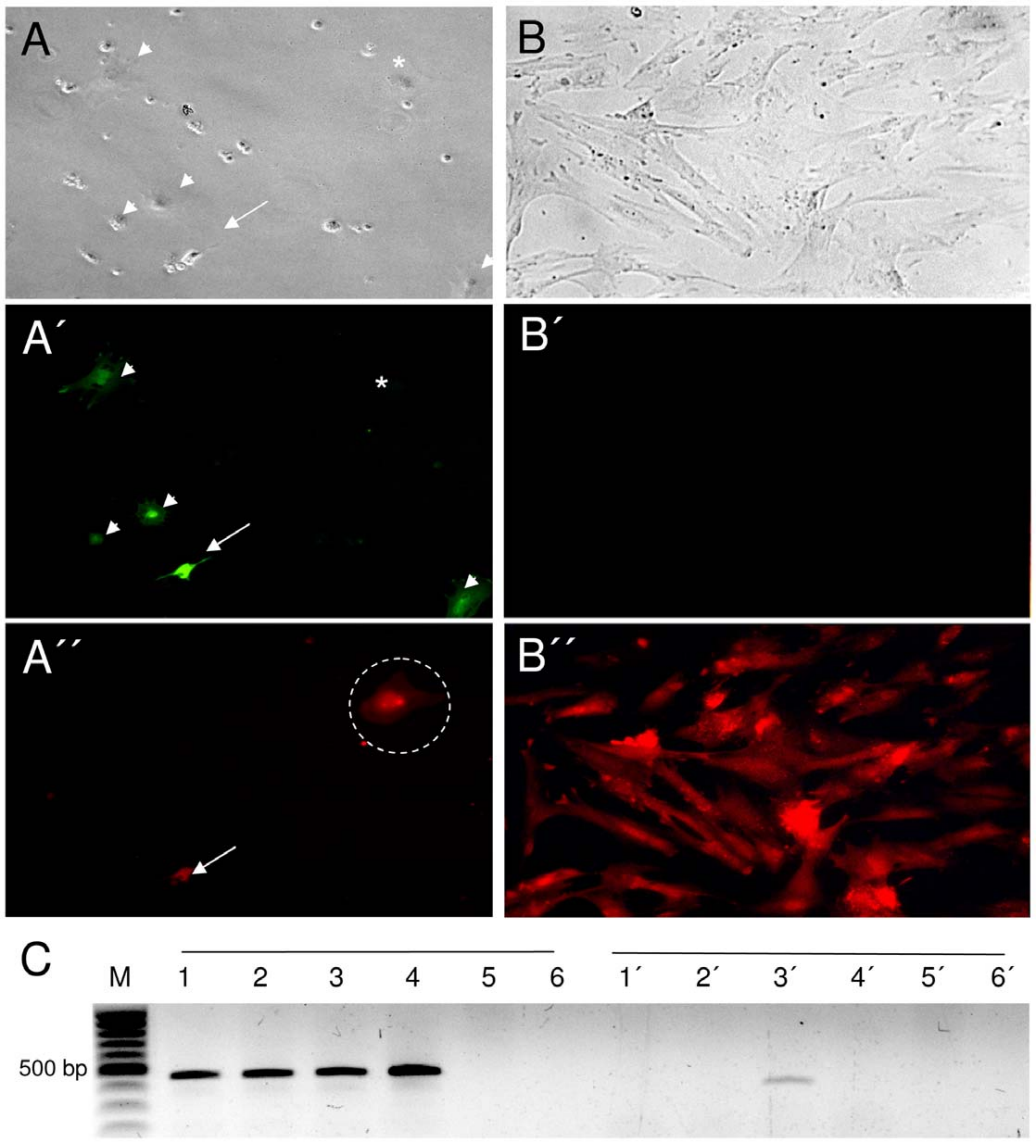
Recombinase-mediated cassette exchange in *Venus*-transposon transgenic fibroblasts. Primary fibroblasts from fetus #37-5 carry a single *Venus*-transposon. The *Venus*-transposon includes heterologous *loxP* sites (see Figure S1). Five day after co-electroporation of a Cre expression plasmid and an *mCherry* exchange plasmid, the cells were screened under brightfield (**A**), *Venus*-optics (**A**′) and *mCherry* optics (**A″**). The dashed circle indicates a cell, which presumptively underwent Cre-mediated cassette exchange (*mCherry* positive and *Venus*-negative). The arrow points to a cell with an illegitimate recombination event (*mCherry* positive and *Venus* positive). Some round cells (most likely dead cells) are floating in the medium and are out of the focus plane and thus do not appear in A′or A″. **B**–**B″**) Clonal isolation and expansion of Cre-recombined cells. Importantly, the screening and clonal isolation procedures are based only on fluorescence criteria and no antibiotics selection was applied. **C**) PCR confirmation of specific cassette exchange. Batch fibroblasts were analysed 10 days after electroporation. In lanes 1-6, primers specific for the *Venus*-transposon (see Fig. 6A for primer positions) were employed (amplicon size 480 bp); in lanes 1′–6′, primers specific for a successful RMCE event (see Fig. 6A) were employed, which specifically amplify a 395-bp fragment. Lanes 1–5 correspond to fibroblasts (#37-5) electroporated with no plasmid (1), with *mCherry* exchange plasmid (2), with *mCherry* and Cre plasmids (3), or were untreated (4), or wildtype fibroblast (5). Lane 6 is a negative control with no template. In lane 3′ an amplicon of the expected size for a successful RMCE event is detectable.

**Figure 6:**
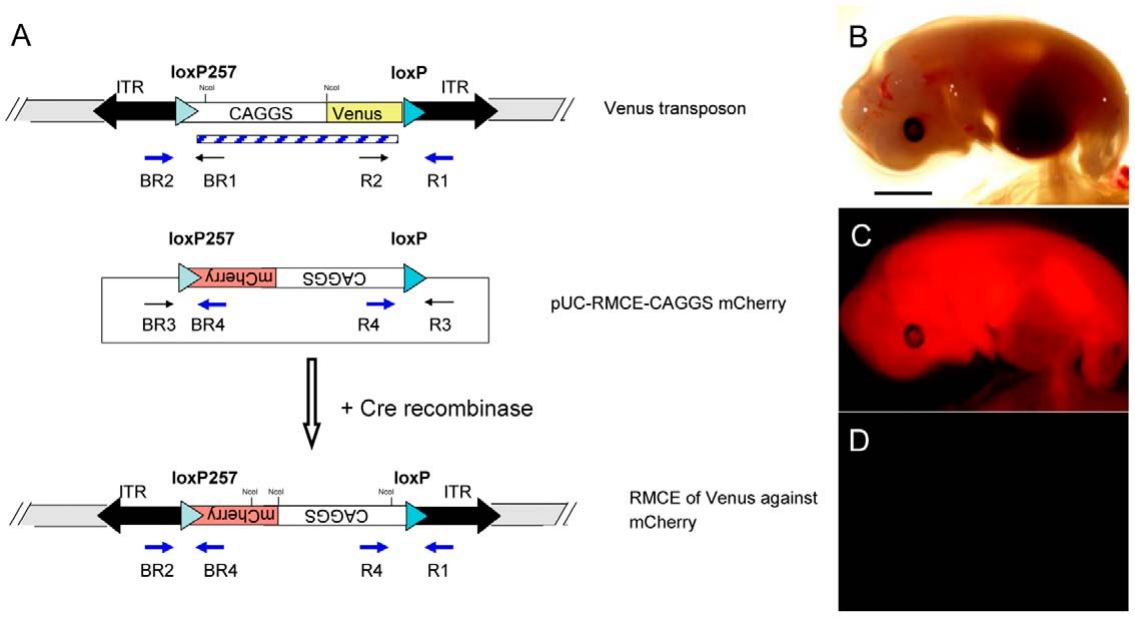
Recombination-mediated cassette exchange in the pig. **A**) Schematic depiction of RMCE in transposon-tagged porcine cells. **B–D**) Fetus obtained from nuclear transfer of RMCE fibroblasts photographed under brightfield (**B**), *mCherry* fluorescence (**C**) and *Venus* fluorescence (**D**) conditions.

The *Venus*-transposon contains heterologous *loxP* sites (Figure S1), which should allow targeted exchange of the *Venus* transgene cassette against a transgene of choice by transient expression of Cre recombinase. Thus, transposon-tagged loci can be retargeted by recombinase-mediated cassette exchange (RMCE) to site-specifically integrate any transgene of interest in a pretested locus. In a proof-of-principle experiment, cultured cells isolated of fetus #37-5, carrying a single *Venus*-transposon, were co-electroporated with a floxed CAGGS-*mCherry* plasmid and a Cre expression plasmid (Figs. 5 and 6A). Five days after electroporation, individual cells were identified, which were *mCherry* positive, but *Venus*-negative (Fig. 5). A total of nine clones were isolated with the expected phenotype suggesting an RMCE frequency of 0.009% in pig fibroblasts. The *mCherry*-positive cells were used as donor cells in a SCNT experiment and a total of 100 reconstructed embryos were transferred to one recipient, which was sacrificed at day 30 after embryo transfer. A total of 12 normally developed fetuses were recovered, all showed specific *mCherry* fluorescence (Figs. 6B and 6C), but no *Venus* fluorescence (Fig. 6D), and a lack of the Cre expression cassette (not shown). Sequencing from both sides of the flanking genomic DNAs isolated from six fetuses confirmed the correct RMCE recombination events (Fig. 6E).

## Discussion

In this work we established the use of the *Sleeping Beauty* transposon system for the generation of germline competent transgenic pigs by cytoplasmic plasmid injection (CPI) into porcine zygotes. An overall transgenic frequency of 6.8% per injected zygotes was achieved, corresponding to 57% and 42% transgenic frequencies in fetuses and born piglets per litter, respectively. The use of the *SB* transposon system for porcine transgenesis has been explored to some extent by other investigators; e.g. by generating primary transposition events and antibiotic selection in fibroblasts that were later used for SCNT [34], [35], or transgenesis was assessed early at blastocyst stage, thereby precluding analysis of transgene expression and germline transmission in founders [35]. Thus, to our knowledge, our work represents the first report on the generation of germline-competent porcine founders by direct microinjection of simple transposon constructs into zygotes. Two of the founders were found to carry a single integrated transposon in their genome. The other founders carried multiple monomeric insertions (2–10), which segregate in the F1 generation. Thus a founder with 3 monomeric insertions can be bred to yield 3 independent transgenic lines, each carrying a single and unique transposon integration. Considering the necessary elaborative resources associated with transgenesis in the pig, this might help to reduce costs and to decrease animal numbers.

**Figure 7:**
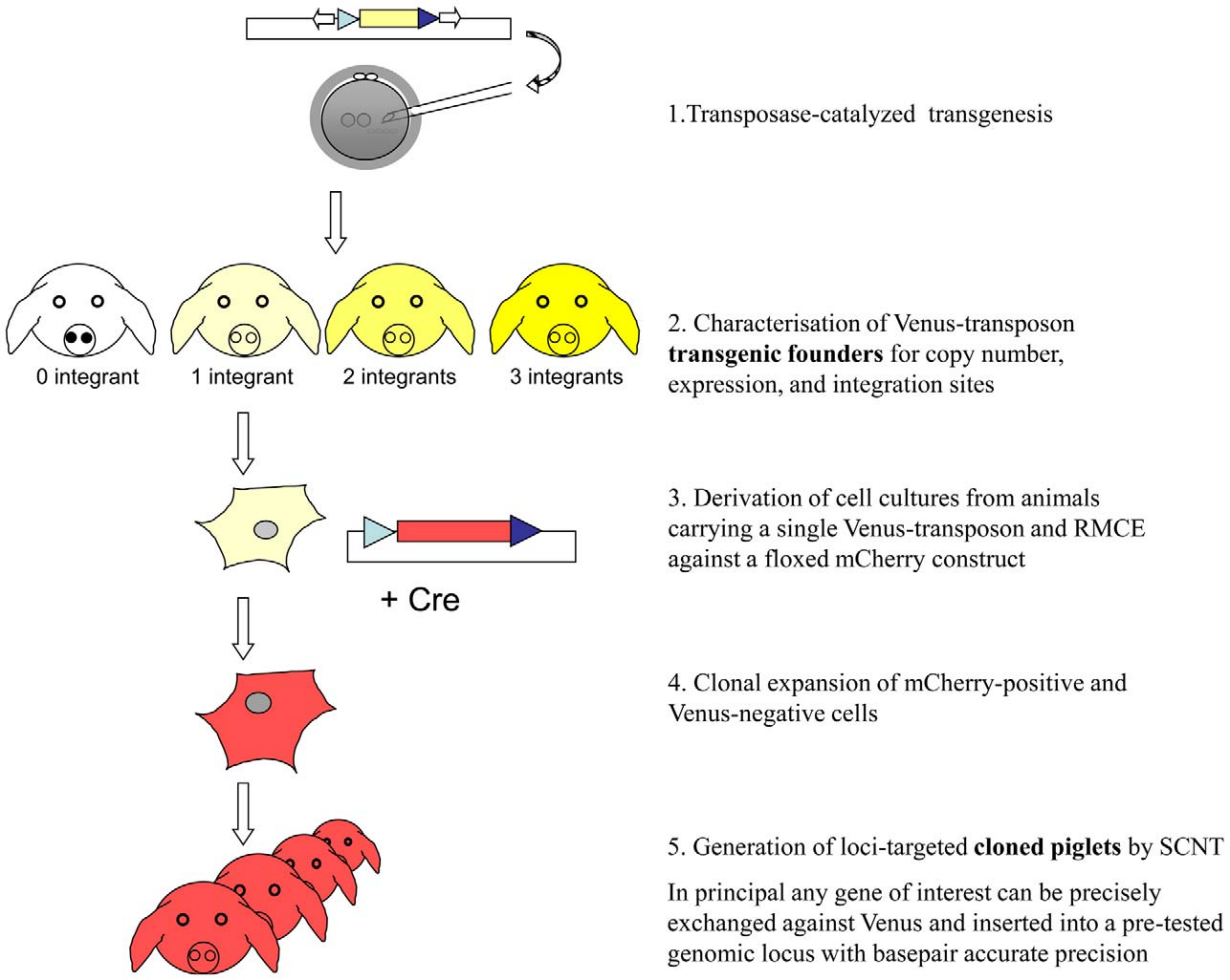
Schematic depiction of targeted transgenes in the pig genome. First, *SB*-catalyzed transgenesis was employed to tag genomic loci, which are suitable for expression. Founder animals with appropriate expression levels and only one transposon integration were selected and used to derive primary cell cultures. Then, the reporter construct (*Venus*) was specifically exchanged against a gene of interest (here *mCherry*) via Cre recombinase and heterospecific *loxP* sites (blue triangles). Cells which underwent successful RMCE events were isolated by screening for loss of *Venus* fluorescence and gain of *mCherry* fluorescence, and were used in somatic cell nuclear transfer to establish cloned piglets, with targeted *mCherry* integration into a pre-tested genomic locus.

In addition, primary fibroblasts of animals carrying a single transposon integration were used for targeted transgenesis by RMCE, and animals were successfully reconstituted from the targeted fibroblasts by SCNT, demonstrating the feasibility of an experimental pipeline of targeted transgenesis into transposon-tagged genomic loci (Fig. 7).

Mechanistically, the classic methods of porcine transgenesis by pronuclear injection and SCNT rely on the cellular repair machinery, which becomes activated by spontaneous DNA double strand breaks (DSB) introduced randomly in the genome by physical and chemical damage of the chromosomes. At a low frequency, foreign DNA may erroneously become integrated into a DSB by the non-homologous end joining pathway [37]–[39]. However, since roughly 70% of higher genomes consist of repetitive elements (telomeres, centromeres, SINEs and LINEs) and other non-transcribed chromosomal structures [38], [39], the majority of DSB-mediated integrations will occur in transcriptionally inactive chromosomal regions. In addition, the preferred substrate for the non-homologous end joining pathway is linearized DNA and, as a consequence, integration of concatemerized gene constructs is commonly observed [37]. Extensive screening of numerous founder animals is currently the only possibility to identify animals with single-copy integrations and the desired expression pattern. The pre-screening of transfected nuclear donor cells in SCNT experiments for transgene and/or selection marker expression is not informative in most cases, since compaction and methylation of genomic DNA change dramatically in cultured cells and the commonly used fibroblasts do not transcribe certain tissue-specific promoters [5], [18].

In contrast, *SB* transposition in microinjected porcine zygotes resulted in genomic integrations of monomeric units of transgenes into the pig genome. Single units of expression cassettes are presumably less prone to transgene silencing than the concatemeric insertions created by classical methods. Indeed, all genotypically transgenic animals (F0 and F1) were also phenotypically positive with no evidence of gene silencing or variegated transgene expression [20], [21]. Our results corroborate and generalize recent data of *SB100X* transgenesis in rodents [33], [40], and supports the hypothesis that *SB100X*-catalyzed DNA integrations preferentially occur in accessible euchromatic regions. Even after long-term observation (more than 12 months), no changes in transgene expression were found in the transgenic pigs, indicating that no age-related silencing of the transgene occurred. Germline transmission and segregation of the *Venus*-tagged transposon was shown by breeding and recovery of F1-fetuses and the birth of healthy piglets. Upon germline transmission, a clear copy number-dependent fluorescence intensity was found in F1-fetuses, indicating that most insertion sites are located in transcriptionally permissive domains of the genome [41]. In line with these observations, Grabundzija *et al.* found that transposon insertions delivered by the *SB* system only rarely (<4% of all insertions) undergo silencing in HeLa cells [41]. Furthermore, stable transgene expression observed in >200 independent insertions in that study suggests that *SB* rarely targets heterochromatic chromosomal regions for insertion, and that it is unlikely that certain sequence motifs in the transposon vector are recognized by mediators of silencing in the cell [41].

Transposition-mediated transgene integration is somewhat analogous to viral transduction by injection of the viruses into the subzonal space of a zygote [42], [43], which results in high ratios of transgenic rodents and farm animals. In this setting, a viral integrase catalyzes integration into the genome; it has been shown that lentiviruses prefer exonic regions of transcribed genes for integration [44], [45]. Thus, an increased risk of insertional mutagenesis is associated with lentiviral transgenesis. The limited cargo, the preferential integration into exons, the occurrence of highly mosaic founder animals and transgene silencing limit the application perspective for lentiviral transgenesis [46]. In contrast, the *SB* transposon has a close-to-random insertion profile in mammalian genomes, and the majority of *SB* insertions occur outside of genes. The majority of the integrations that occur within genes are localized in introns [47]–[49], and the integration sites in the pig genome appear to follow the same rules.

The molecular events and timing of transposition in porcine embryos need to be further investigated. Previous experiments suggested that CMV promoter-driven marker genes in circular plasmids are transcribed concomitantly with major embryonic genome activation [36], which is at the 4-cell stage in porcine embryos [50]. How the circular plasmids translocate from cytoplasm to nucleus is currently unclear, a possibility is that some plasmids enter the nucleus region after nuclear membrane disassembly during the first cell cycle.

The majority of founder pigs generated here seem to express Venus homogenously. A molecular analysis of the founder boars #503 and #505, however, suggested that albeit all analyzed tissue biopsies (ear skin and sperm) were transgenic, the integration sites seem to differ between tissues. To clarify, whether this may be due to late integration events in different blastomeres, or to re-mobilization of early integrants by the transiently present SB protein requests a more detailed study. Finally, we demonstrate targeted transgene integration into genomic sites that are defined by *SB* transposon insertions carrying heterospecific *loxP* sites by transient expression of Cre recombinase in the presence of a floxed *mCherry* construct. Thus, transgene cassettes of interest can be serially knocked into the exact same position in the genome allowing comparative gene expression studies. Fibroblasts with transcriptionally permissive chromosomal loci tagged by *SB* transposon insertions can thus be utilized as master clones amenable to advanced genetic engineering in the pig. In conclusion, the technology based on cytoplasmic microinjection of *Sleeping Beauty* transposon plasmids is a simple and efficient technique yielding transgenic pigs with germline transmission and stable transgene expression at efficiencies that improve genome modifications in the pig, and thus may allow generating better models for human diseases.

## Materials and Methods

### Ethics statement

Animals were maintained and handled according to the German guidelines for animal welfare, and to the German law regarding genetically modified organisms. The animal experiments were approved by an external ethics committee (Niedersächsisches Landesamt für Verbraucherschutz und Lebensmittelsicherheit, AZ 33.9-42502-04-09/1718).

### Animal experimentation: Superovulation, flushing of zygotes, embryo transfer (ET), recovery of fetuses, sperm analysis, artificial insemination

Gilts were superovulated by intramuscular injection of 1'000 U Intergonan/PMSG (96 h before insemination) and 500 U Ovogest/hCG (24 h before insemination) and then artificially inseminated on day 0. The next day, the animals were slaughtered, oviducts were excised, flushed with PBS/1% new born calf serum and zygotes were collected and used for microinjection. 30–40 intact embryos were surgically transferred into the oviduct of a synchronized recipient. In some cases, pregnant recipients were sacrificed and fetuses were recovered. Sperm cells were obtained from wildtype and transgenic boars using a phantom and immediately diluted in Androhep solution.

### Preparation of ccc-plasmids and cytoplasmic injection

The plasmids pT2/RMCEVenus and pCMV-SB100X were transformed in XL10 or ER2925 bacteria, respectively. Supercoiled plasmid DNA was isolated with anion exchange columns and resuspended in ultrapure water and checked for the absence of bacterial genomic DNA or endotoxins [36]. The DNA concentration was determined by a NanoDrop photometer; purity and supercoiled ccc-conformation were verified by gel electrophoresis. Plasmids were prepared in 10 mM Tris-HCl pH 7.6 and 0.25 mM EDTA pH 8.0, and backfilled in glass injection capillaries. Individual embryos were fixed by suction to a holding pipette, while the injection capillary was pushed though Zona pellucida and cell membrane. Approximately 10 pl of the plasmid solution was then injected with a pressure of 1 bar into the cytoplasm using a pressure-controlled Eppendorf transjector 5246 (Eppendorf, Hamburg, Germany). SB100X-mRNA was prepared as described [33].

### Fluorescent microscopy and macroscopic excitation of Venus fluorochrome

For fluorescence microscopy, a Zeiss Axiovert 35 M microscope equipped with fluorescence optics for Hoechst 33342, GFP and rhodamine was used. Alternatively, images were obtained by an Olympus BX 60 (Olympus, Hamburg, Germany) fluorescence microscope equipped with a high resolution digital camera (Olympus DP71).

### Genotyping and identification of integration sites

Transposon-genomic DNA junctions were determined using splinkerette PCR as described earlier [22]. The purified PCR product was cloned into the pGEM-Teasy vector (Promega, Madison, USA), and the DNA sequence was determined by standard sequencing technology (ABI3730XL Applied Biosystems, Foster City, California). Sequences were analyzed with BLAST at www.ensembl.org (assessed 24.08.10). Southern blots and PCR reactions of genomic DNA were done according to standard procedures. In brief, for Southern blot detection of Venus transposon copies, the genomic DNA was digested with NcoI. Hybridisation with a *CAGGS-Venus* probe (1.4 kb fragment generated by EagI digest of pT2/RMCE) then resulted in a constant internal fragment of ∼1.4 kb and a variable external fragment of >1.4 kb per integration (Figure S1). To assess for the presence of random integrations of the transposon, the genomic DNA was digested with BamHI. On the transposon plasmid the ITRs are flanked by 2 sites for BamHI in neighoring sequences. In case of SB100X catalyzed integrations these BamHI sites are lost. Unspecific integrations should produce a constant 3.39 kb fragment after hybridization with the *CAGGS-Venus* probe (Figure S1). However, no indications of unspecific integrations were detected (not shown). To assess for *SB100X* sequences, the blots were hybridized with a *SB100X* probe, generated by labeling the whole plasmid.

### Preparation of primary cell culture and FACS measurements

Primary cells were derived from fetal and adult tissues as described [21] and cultured in DMEM supplemented with 10% fetal calf serum and antibiotics. Leukocytes were isolated from EDTA-blood samples and resuspended in PBS. Flowcytometry analysis of primary cells, leukocytes and spermatozoa was performed using a FACScan (BD Bioscience, Heidelberg, Germany) equipped with an argon laser (488 nm, 15 mW). Samples were diluted to 0.5×10^6^ cells/ml and measured in dublicates acquiring 10 000 cells per sample. Cells with membrane damage were excluded from the analysis by counterstaining with propidium iodide (20 µM).

### Recombination-mediated cassette exchange (RMCE) in primary fibroblasts

300 000 fibroblasts (passage 2) isolated from a fetus, which carried a single floxed Venus-transposon (Figure S1) were co-electroporated with 200 ng of pCAG-Cre (gift from Dr. C. Cepko via www.addgene.org) and 500 ng of pUC-RMCE-CAGGSmCherry, carrying a floxed mCherry expression cassette. For electroporation rectangular pluses of 100 V and 10 ms (Biorad, GenePulserxcell, München, Germany) were applied and the cells were seeded in Petri-dishes. Cell clones which only expressed mCherry, but were Venus-negative, were subcloned by cloning cylinders.

### Northern blot

Total RNA was extracted from tissues using TRIsure Reagent (TRIsure; BIOLINE GmbH, Luckenwalde, Germany) according to standard procedures. RNA quality was evaluated with a 2100 Bioanalyzer (RNA nano chip, Agilent Technologies, Waldbronn, Germany). Northern blotting was performed as described [24] and blots were hybridized overnight (68°C) with DIG labeled probes complementary to EGFP (900 bp) and subsequently to ACTB. Hybridization signals were detected by chemiluminescence (Fusion FX, Vilber Lourmat, Eberhardzell, Germany).

### Somatic nuclear transfer of RMCE cells

Somatic nuclear transfer was performed as described recently [51]. In brief, oocytes were enucleated by removing the first polar body along with the adjacent cytoplasm containing the metaphase plate. A fibroblast from the RMCE cells was placed in the perivitelline space in close contact with the oocyte membrane. Cell membran fusion was induced in Ca^2+^-free medium (0.25 mol/l sorbitol, 0.5 mmol/l Mg-acetate, 0.1% BSA) by a single electrical pulse of 1.1 kV/cm for 100 µs (Eppendorf Multiporator, Eppendorf, Germany). The reconstructed embryos were activated in an electrical field of 1.0 kV/cm for 45 µs in SOR2 activation medium (0.25 mol/l sorbitol, 0.1 mmol/l Ca-acetate, 0.5 mmol/l Mg-acetate, 0.1% BSA) followed by incubation with 2 mmol/l 6-dimethylaminopurine (DMAP, Sigma-Aldrich, Germany) in NCSU23 medium for 3 h prior to embryo transfer to recipients. 100 reconstructed embryos were surgically transferred into the oviducts of one synchonized peripubertal German landrace recipients via mid ventral laparatomy under general anaesthesia. A pregnancy was confirmed by ultrasound scanning on day 25 of gestation, and the recipient was sacrificed and fetuses were recovered at day 30.

## Supporting Information

Figure S1 ***Sleeping beauty*** **plasmids.** The non-autonomous transposon components pCMV-SB100X (Cytomegalovirus promoter (CMV) driven SB100X-cDNA) and pT2/VenusRMCE (CAGGS promoter driven Venus-cDNA flanked by SB internal terminal repeats (ITR)) are co-injection into the cytoplasm of porcine zygotes. After expression of the SB100X plasmid, the SB100X transposase catalyzes transposition of the Venus transposon to a chromosome, thereby creating a stably transgenic status. Backbone sequences of the Venus-plasmid and the SB100X-plasmid are lost or become degraded over time. In the absence of SB, the Venus transposon is fixed at a certain integration site. Hatched bar indicate the Venus-specific probe for Southern blotting, genomic DNA was digested with NcoI, thus labelling of an internal fragment of ∼1.4 kb and an external fragment(s) of >1.4 kb is expected. For the detection of unspecific integrated Venus constructs, the genomic DNA was digested with BamHI. The BamHI sites flanking the Venus transposon on the pTE/VenusRMCE plasmid are lost during specific transposition. Backbone and SB100X-specific probes were used to assay for presence of these sequences by Southern blotting. Arrows indicate the positions of primers used for PCR genotyping. Blue triangles stand for heterospecific loxP sites. Drawing is not at scale.
(TIF)

Figure S2 **Alignment of identified integration site to porcine chromosome X.** Exemplarily, the integration sites on the porcine chromosome X is depicted (No. 3 clone 37_2_2 in Table S2). The integration site was aligned to the porcine genome by the ensembl resource (www.ensembl.org). The displayed area covers a 20 kb stretch of chromosomal DNA, and the location of transposon integration (red bar in line labeled BLAT/BLAST hits), as well as the positions of annotated or predicted genes, LTRs, retrotransposal elements, and G/C ratio are shown.
(TIF)

Figure S3 **Southern blot hybridized with backbone probe.** The blot shown in Fig. 2E was stripped and hybridized with a backbone probe of pT2/VenusRMCE. The backbone probe was generated by BamHI digest of pT2/VenusRMCE and isolation of a 2.8 kb fragment. A randomly integrated backbone sequence should result in a >2.8 kb fragment after Southern blotting.
(TIF)

Table S1 **List of transgenic founders and F1-offspring.**
(DOC)

Table S2 **Transposon integration sites in the pig genome.**
(DOC)

Video S1 **F1-litter of Venus-fluorescent piglets.** Breeding of founder boar #503 with a wild-type sow resulted in a litter of eight piglets. Founder #503 carries three single copy transposon integrations. The calculated ratio of transgenic offspring is 87.5% (permutations of the three integrants), if the integration sites represent indeed single copy events and are independently inherited. The piglets are shown under illumination with (i) normal light, (ii) blue light excitation and (iii) blue light excitation and emission filter. Note the different fluorescence intensities at specific excitation (condition iii). The fluorescence intensities directly correlated with the number of transposon copies as determined by Southern blotting. The two non-transgenic piglets are only vaguely visible under fluorescence recording.
(WMV)
