# Supplementary figures and images for: Germline Transgenic Pigs by Sleeping Beauty Transposition in Porcine Zygotes and Targeted Integration in the Pig Genome
Source: PLoS One. 2011 Aug 29;6(8):e23573. doi: 10.1371/journal.pone.0023573 (PMC3163581; doi:10.1371/journal.pone.0023573)

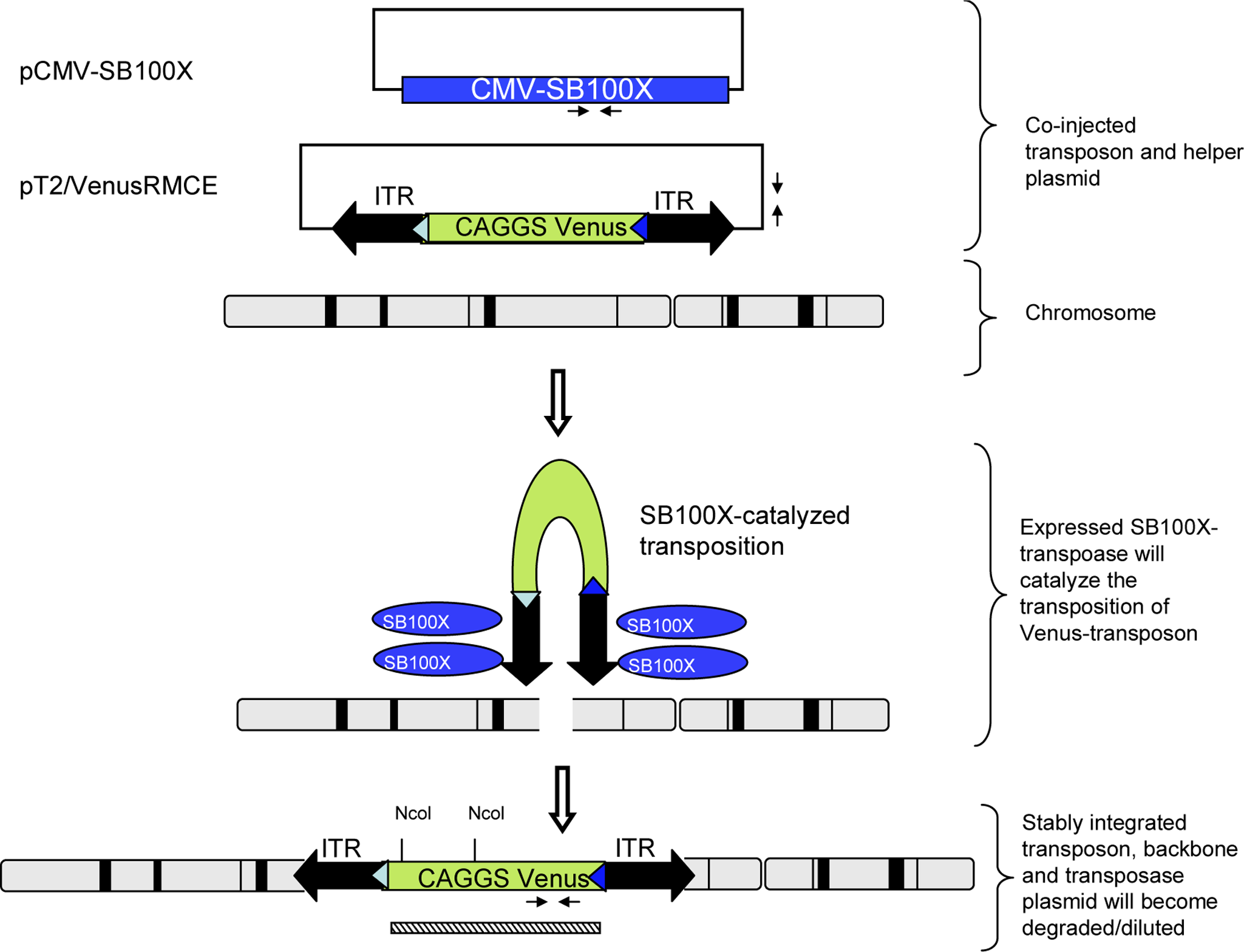

Supplement: Figure S1 — Sleeping beauty plasmids. The non-autonomous transposon components pCMV-SB100X (Cytomegalovirus promoter (CMV) driven SB100X-cDNA) and pT2/VenusRMCE (CAGGS promoter driven Venus-cDNA flanked by SB internal terminal repeats (ITR)) are co-injection into the cytoplasm of porcine zygotes. After expression of the SB100X plasmid, the SB100X transposase catalyzes transposition of the Venus transposon to a chromosome, thereby creating a stably transgenic status. Backbone sequences of the Venus-plasmid and the SB100X-plasmid are lost or become degraded over time. In the absence of SB, the Venus transposon is fixed at a certain integration site. Hatched bar indicate the Venus-specific probe for Southern blotting, genomic DNA was digested with NcoI, thus labelling of an internal fragment of ∼1.4 kb and an external fragment(s) of >1.4 kb is expected. For the detection of unspecific integrated Venus constructs, the genomic DNA was digested with BamHI. The BamHI sites flanking the Venus transposon on the pTE/VenusRMCE plasmid are lost during specific transposition. Backbone and SB100X-specific probes were used to assay for presence of these sequences by Southern blotting. Arrows indicate the positions of primers used for PCR genotyping. Blue triangles stand for heterospecific loxP sites. Drawing is not at scale. (TIF) [file pone.0023573.s001.tif]

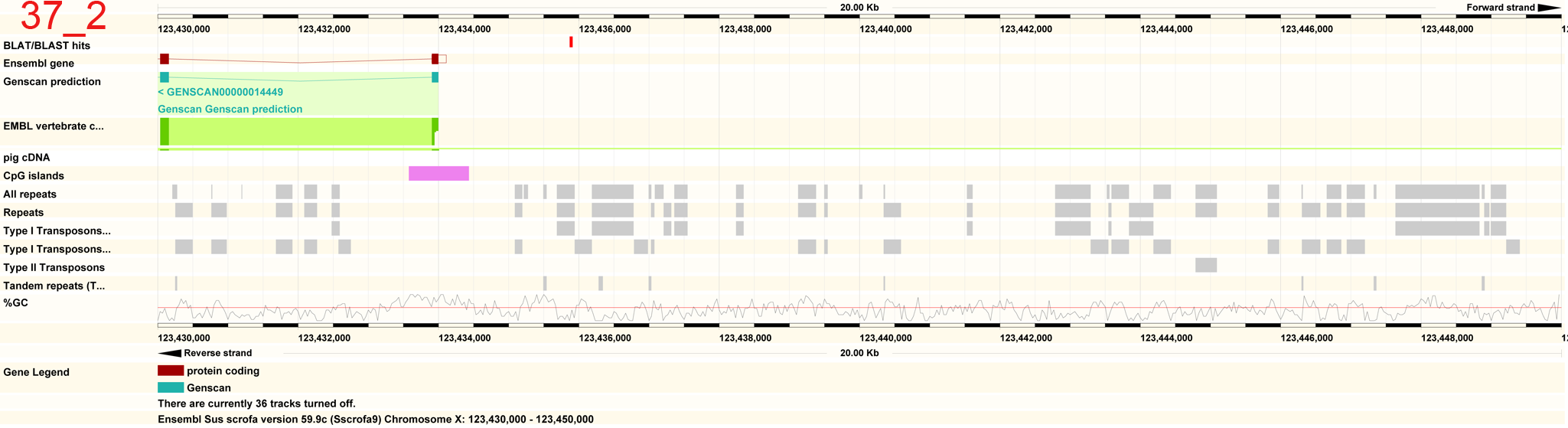

Supplement: Figure S2 — Alignment of identified integration site to porcine chromosome X. Exemplarily, the integration sites on the porcine chromosome X is depicted (No. 3 clone 37_2_2 in Table S2). The integration site was aligned to the porcine genome by the ensembl resource (www.ensembl.org). The displayed area covers a 20 kb stretch of chromosomal DNA, and the location of transposon integration (red bar in line labeled BLAT/BLAST hits), as well as the positions of annotated or predicted genes, LTRs, retrotransposal elements, and G/C ratio are shown. (TIF) [file pone.0023573.s002.tif]

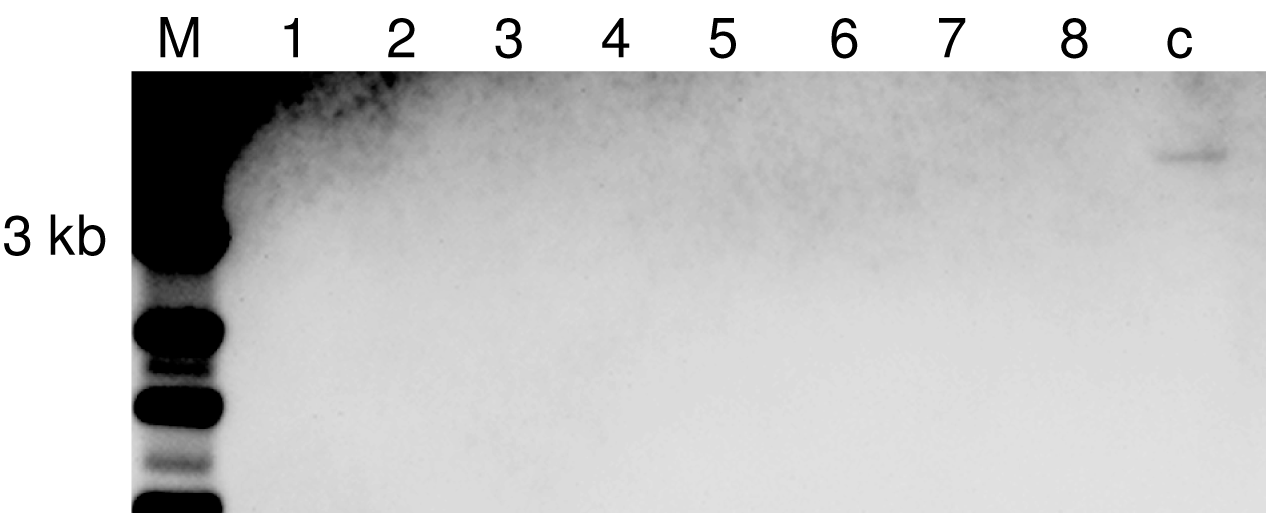

Supplement: Figure S3 — Southern blot hybridized with backbone probe. The blot shown in Fig. 2E was stripped and hybridized with a backbone probe of pT2/VenusRMCE. The backbone probe was generated by BamHI digest of pT2/VenusRMCE and isolation of a 2.8 kb fragment. A randomly integrated backbone sequence should result in a >2.8 kb fragment after Southern blotting. (TIF) [file pone.0023573.s003.tif]
