# Supplementary material for: Germline Transgenic Pigs by Sleeping Beauty Transposition in Porcine Zygotes and Targeted Integration in the Pig Genome
Source: PLoS One. 2011 Aug 29;6(8):e23573. doi: 10.1371/journal.pone.0023573 (PMC3163581; doi:10.1371/journal.pone.0023573)
Supplement: Table S1 — List of transgenic founders and F1-offspring. (DOC) [file pone.0023573.s004.doc]

Supplementary Table 1 List of transgenic founders and F1-offspring

| Status | F | Individual animals | Venus expression (phenotype) | Copy number of Venus-transposon | Presence of helper plasmid or Venus plasmid backbone |
| --- | --- | --- | --- | --- | --- |
| Founder (Fetuses) | F0 | #37-1  #37-2  #37-3  #37-4  #37-5  #40-1  #40-2, degenerated | -  +  +  +  +  only amnion +  n.a. | none  >10  ~ 5  3  1  n.a.  n.a. | -  -  amnion SB+  -  -  -  - |
| Born founder | F0 | 1 = #505  2 = #503  3  4  5  6  7  8  9  10  11, stillborn  12, stillborn | +  +  -  -  -  -  +  +  -  -  -  + | 3  3  0  0  0  0  2  2  0  0  0  1 | -  -  -  -  -  -  backbone +  -  -  -  -  backbone + |
| Fetuses of filial generation  (father #503,  three pregnancies) | F1 | F1-1  F1-2, degenerated  F1-3  F1-4  F1-5  F1-6, degenerated  F1-7  F1-8  F1-9  F1-10  F1-11  F1-12  F1-13  F1-14  F1-15, degenerated  F1-16  F1-17  F1-18  F1-19  F1-20  F1-21 | +  +  ++  ++  ++  n.a.  +  ++  ++  +  +  ++  +  ++  n.a.  ++  -  +  +  ++  - | 1  1  2  2  2  n.a.  1  2  2  1  1  2  1  2  n.a  3  0  1  1  2  0 | -  -  -  -  -  n.a.  -  -  -  -  -  -  -  -  n.a  -  -  -  -  -  - |
| Born filial generation  (father #503,  one litter) | F1 | F1-22  F1-23  F1-24  F1-25  F1-26  F1-27  F1-28  F1-30 | ++  +  ++  -  ++  ++  -  ++ | 2  1  2  0  2  2  0  2 | -  -  -  -  -  -  -  - |

n.a., not applicable
