# Supplementary material for: Germline Transgenic Pigs by Sleeping Beauty Transposition in Porcine Zygotes and Targeted Integration in the Pig Genome
Source: PLoS One. 2011 Aug 29;6(8):e23573. doi: 10.1371/journal.pone.0023573 (PMC3163581; doi:10.1371/journal.pone.0023573)
Supplement: Table S2 — Transposon integration sites in the pig genome. (DOC) [file pone.0023573.s005.doc]

Supplementary Table 2. Transposon integration sites in the pig genome

| No | Clone No. | Sequence | Alignment | Coverage (bp) | Identity (%) |
| --- | --- | --- | --- | --- | --- |
| 1 | 37_3_2 | TAGATTCTTCTTTTG | no hit |  |  |
| 2 | 37_3_4 | TATTTATTCTTTGCTTCTGGAGCTAATAG | no hit |  |  |
| 3 | 37_2_2 | TATGTCTATTTTCTCCAAATTTAACCATAGATTCAATGTA | **Chr. X,**  123435870-123435909 | 40 | 100 |
| 4 | 37_2_6 | TATCTCTGGATTTTTT | no hit |  |  |
| 5 | 37_1_12 | TATGTGTATGTGTGGTGTGTGTGTGTGGACATGTGTGCCTGCGTGTACACCTGTGTGTGCATGCATGTGTATGTGTGGGTGCATGCATGTCTGTATGT | no hit |  |  |
| 6 | 37_4_153 | TATGGTTTACTGATTTCTGACCCTAAATATGTGATTCTCTCCAGAGTTGCCTATCTCATTGATACCTGGAATCATGTCAGCAGGGTAGGAGTGCCTCTTATTTCAAGACTCAAATTTAAATGACACTATTTCAAAGAGAACACGCTATATTTATTATGTAATATATTTGTAGCT | **Chr.13,**  122989199-122989372 | 174 | 99.4 |
| 7 | 37_4_155 | TAATACCAACTTAAGACAGACTGGATTGGGGTTCCTGCTGTGGTTCGACAGGTTAAGGACCCTCTCTGAGGACGGGGGTTCGATCGTCCCTTAAGCGGAGCCCT | partial alignment, Chr.12 | 85 | 96.5 |
| 8 | 37_4_157 | TATTCAAAGTTCTAAAAGGGAAGAATTTGCAGCCTAGAATCCTATACTCAGCA | repeat seq.  Chr.X, 6,7,13 |  |  |
| 9 | p1_1 | TATCCTGGAGACGTTTTGCTTACAAACGCTCTCACCTTAATAGATAGCGTGAAAAAATCTAATCTATCTCCAGTAATTAAAAAATCAATAATGTATTAT | **Chr.3,**  119120423-119120522 | 99 | 99.0 |
| 10 | p1_4 | TACACAGTCTAATTTCAGGCAGTGATGAGTATTATAAAGGATATAAAACAGTGATGGAACAAAGCGTGATGGAGTTCCCGTTGTGGCTCAGTGGTAACAAACCCGACTAGCATCCATGAGGACACGGGTTCCATCCCTGGCCTTGCTCCATGGGTACAGGTACAGTGTTGCCTTGAGCTGTGGTGTAGGTCGCAGTCACATCTCG | repeat seq.  Chr.3,11,12 |  |  |
| 11 | p1_5 | TACCAAGTCATACACCTCAACAACAACAACAAACGACCCAATTGAAAAATGGGCAGAAGTCCTAAATAGACATTTCTCCAAAGAAGACATACGGATGGCCAGTAGGCACATGAAAAATTGCTCAACATCACTAATTAGCAGAGAAATGCAAATCAAAACTACTATGAGGTACCACCTCACACGAGTCAGTATGGCCATCATTAATAAGCCTACAAATAACAAATGCTGGAGAGGGTGTAGAGAGAAGGGAACCCTCCTACACTGTTGGTGAAAATATAAATTCAGGATTTCCCATCGTGACGCAGTGGTAATTAATCTGACTAGGAACCATGAGGATGCAGGTTCAATTCCTGGCCTCCCTCAGTGGGTTAAG | **Chr. 7,**  27601184-27601559 | 373 | 99.2 |
| 12 | p1_6 | TATATATATTTTTATGGACCTTGTGTGAATCCCGATACCAACTATAAAAAGTTACTGCTGGGATAACTAGGGAAATTTGAATATAAAATAGGTAACAGATGGTATTAAGTAAATCGTGTTCATTCTTTGAGTTAGACATGTTCAAAGTGGTATGGAAGATATGCTTTTTTTAAAAAAATCTTTTCGTGTCGAAGATC | no hit |  |  |
| 13 | p1_3 | TATCAGGAAGGCACATGGTGACAGTGTTTCACTGAGTCACAAACGTAGCCCAATCCATACAACTAACAACGAGCTGTCAATAGGCTTCTTCCTAGTTATGTCCTACAGATGACAAGCTTCAGTTGTTTGAACTGAGGCGCATATATCTACACATATTTTGAACAATTTCGTTTCTTAGTACATATTAATAAAAGCCAAAAACCTGCAATGAGAAGCTATTGGGGCTTAAAAAAAGACTGTTCTTTTATTGTGATTATGTTTGGATCGTCCCTTAAGCGGA | **Chr.8**  72679733-72679998 | 266 | 99.3 |
| 14 | p2_1 | TACGAAGCGGCCTTAATCTGAATCGTGGGGAAATATATCTTGGGCCAAAACTGATGCTCTCTACTGGGGCCTACCCTTGCGCGCGTCCTATAAAAGGTCCGGGTGGTGAAACTGCCCGGCCCCTGGACAGAGCTCCACTCATACCTGATTTATGGCTCTATCCCCAACGTTTCATCGCCTGTATTGTTTATTTACGCTACCAAATCATCCGCCTGGATATCTTATACCTGCTTGGTTATACTCTGTAGCACAGATCCTGCAACTGGATCTCTATTCAATGTTCTTTATCCTTCGTTACTACAGTTATGTCAATTAACTTTTTTATTACCCACCACTCACATCCTCTAAGGGACCACCGATCCGCTGGCTCTTATTACAAAGCCGGAAATCGGTCCCGGAGAAAAGGGAACCCTCCGTCCACTGTTGCTGTTCTGCATCATAAAGGGAAGATTCTCTCTCCGCCGTTGTAATTTGATATGGTCGTTTCCTCAGAAAACTCATTATTATCTACCAATGTCCTCTGTCGTTCATTTTCGTGTTTATTCTTGTGCTTTATCCCAGAATCTCGCCAACAAC | ambiguous |  |  |
| 15 | p2_3 | TACATGCGGCTTGGATCTGAATGGCTCTTGAGAATATCTTGCGCCAAAACTGTTGCACTCTAATGTCGACTACACTTGCTTGTGACCTCTCTTGGTCCGGCTGGAGGAACTAAACGGCTCCTGACAAAGAAGGACACTACTAACTGATACATTGCTCTGCTTTCAACGCTTGATCGCTTGGCTCTAAATTTTTTGTAAGCAATATCCCAAAAAAAAATGGTATGGCACCTAAATCAATATTTCTCCCAGAAATGCCTCCAACAGGATTTCAGTTCTTTGACTAACATACTTAGTGATTACAGAAATGCCAATTAAATTTTTTGTGACCTACCTCACACCTCTCTAAATGACAATTGATCCTAAGGCTACATATAACAAATACTGAAAGGGTGTCGTAGAAAAGGGAACCCTCCTTCACTGCTGGTGGGGTGTGGAGAAAAGGGAACCATCCTTCACTGCTGGTAGAAAGCATTATGGTGGTTCCTCAGAAAACTAAATATAAAACTACCATATGACTGTTTGGCCTCGTTTGAAAATCACAAGCATGC | repeat seq.  Chr.7,12,15 |  |  |
| 16 | p2_4 | TAGAGGCGGCTTGGATCCCAGTATCCCCTGGCTCAATTCTCACTGACTTATCACCACACTGAGAAATATAGATTAGAATTTTTTACAAGCTAAGACTTCCCCTGGTCTAGAGAAAGCAGATGTTTTAAGGTGACTTTTGAGGGGCCCCATAACTTCTCTGCTGTTAAAAAACAAACAAAAACACCACTAGCATAAACTATAAAAAGAAGTAAATTCAGCTGAAGGAGACTGTGGGAGGATTGGCCTGCCACCCAGATGATCCCTTGGCTCGTTTTTTTTTGCAAAAAGATCCGAGCCGCATCTGCAACCTACACCACAGCTCACGGCAGCGCTG | repeat seq.  Chr. X,1,3,9, 11 |  |  |
| 17 | p2_5 | TAGATGCGGCTTG | too short |  |  |
| 18 | p2_3 | TAGATGCGGCTTGGATC | identical to #17 |  |  |
| 19 | p2_2 | TACATATAGATTGAAAGTGAAAGGGTGGGGAAAAATATTTCACGCCAATAGACATGACAGAAAAGCAGGAGTCGCAACGCTCATATCAGACAAAATAGACTTTAAAACAAAAGACATAAAGAAAGACAAAGAAGGACACTACTTAATGATTAAGGGATCGTCCCTTAAGCGGAGCCCTAATCACTAGTGCGGCCGCCTGCAGGTCGACCATATGGGAGAGCTCCCAACGCGTTGGATGCATAGCTTGAGTATTCTATAGTGTCACCTAAATAGCTTGGCGTAATCATGGTCATAGCTGTTTCCTGTGTGAAATTGTTATCCGCTCACAATTCCACACAACATACGAGCCGGAAGCATAAAGTGTAAAGCCTGGGGTGCCTAATGAGTGAGCTAACTCACATTAATTGCGTTGCGCTCACTGCCCGCTTTCCAGTCGGGAAACCTGCGTGCCAGCTGCATTAATGAATCGGCCAACGCGCGGGGAGAGGCGGTTTGCGTATTGGGCGCTCTTCCGCTTCCTCGCTCACTGACTCGCTGCGCTCGGTCGTTCGGCTGCGGCGAGCGGTATCAGCTCACTCAAAGGCGGTAATACGGTTATCCACAGAATCATGGGATAACGCAGAAAGAACATGTGAGCAAAAGGCCAGCAAAAGGCCAGGATCGTAAAACGCGCGTGCTGGCGTTTTCCATAGCTCGCCCCTGACGAGCATCACAAAATCGACGCTCAGTCAGAGTGCGAACCCGACAGACTATAAGATACAGGCGTTTCCCCTG | repeat seq.  Chr. X, 1,2, 3, 13,16 |  |  |
| 20 | p7_6 | TATTCTTTCTCTAAAACCTTGTATAAATAGGACTTACCTGGTAGCTCAGTGGGTTAATGGCCTGGCATTGTCACAGGTTTGATCCCTTGGCTCGTTTTTTTTTGCAAAAAAGAGGGATTGCTGGATCATATGGTAGTTCTATATTTAGTTTCTGAGGAATCTCCGTATGGTTTTCCACAGTGACTGTACCAATTTATATTCCCACCAGCAGTGTAGGAGCATTCCTTTTTCTCCACACCCTCTCCAGCATTTATTACTTGTAGACTTCTTAATGATGGCCATTCT | repeat seq.  Chr. 1, 2, 6, 13 |  |  |
| 21 | p7_8 | TACTTTCATTCATTACATTGTCAAAAGGTGAAAAGAGAGATCACTGCCAGGGATGGTTAATAGGCGGTGGTAGCTGGATACTCCAGCAGGAGCCCCCATTCACAATGAGACCTGTGTGGTTAGAGCAGAGAATTTGCCCTTTCTGCCAGGGGAGGGGGAAACCTTTGCTGGGAATTCTAACTCTTTACAACATAGGACATCAGGTAATGAAAAGCAGTTGATGTACTAATTACTGATCCCTTGGCTCGTTTTTTTTTGCAAAAACTCAGGTAAAAAGTTTCACCAAGGAAGCTCTAAAATCAATTGTGCCCAGAAAATCCTTAAGCTGAAA | multiple hits,  Chr. 1, 5, 9 |  |  |
| 22 | p7_4 | TACTTGGATTCCTTACATTGTCAAAAGGTGAAAAGAGAGATCTCTCCAGTGCTAATAAGGATGTGGAGAAATGAGCACACTTAACTATAGCTACTGGAGTTCCCCTTGTAGCTCAGTAGTAAAGAGCCGAAACCCATAGGGTGGGGTTCAATCCCTGGCCTCACTCAGTGGGTTAAGGGCCTTGTTTTGCCATGAGCTGTGGTGTAGGCCACAGATGCAGCTCG | repeat seq. |  |  |
| 23 | p8_2 | TATTACTGATGGTTCTAATTTTCCCTGTGCAAAGGGTGCTCAAAAAGCATGCGGAGGCCCCATACTGAATTCTCTGGTGGACAGCACTGCTTGTCTTGGCCTTGTTCTGACCCCCATGTTTCTCACGTGACAAACCTCACACAGCCCAGCAATGTTACCCCCGGTTCTGAAACAAATGTTCAAATTTGCTGGGTGTCCTCCCCAACCCCAAATTTCACGTGAAAGCCCTGACCCCCAGACCCTCGCAGTGTGATGCTACATATGTATTATTCCTTTACGTGAAATCCTAATACTCGCGGCTATGGTAGCCCAGTAGCATGCAGGGTCGGGCCTAATTCGAGCTAGAGAGTGTATCACTACTATTCTTCGTCCCTCTTTATATATCCCTCA | no hit |  |  |
| 24 | p12_1 | TAAACTCTCACTTCTGCTTTAACCACATTCCATCCAAGTTGATAGGCATTGTTGCACTGCCATGAATTTTTAAATGGCCTGTAATTATGGTACTGATTTCCTCTTTGACTCAAAAACTTATTACAGTTTTTGTTTTAATTTCCAAGGAAGTGGGTATTTTAAGCATTTATAATCATTACTGTTATTATTTGCTTTGTGATCCCTTGGCTCGTTTTTTTTTGCAAAAAAAAATGGGCAAAAGACCTGAATAGTTGAATGAACTCGAAGTCAAAACTTATCTTCTGTGCCATCTCTCTCAGTATGAAGCTGTGTTAATTCCTAGAATTTTCTAGATCTGCAGACGCCGACCTTGGAGCCTTAGGGGTCCATTGT | partial alignments  Chr. 2, 3 |  |  |
| 25 |  | TAAATGTGCACTTGTGCATACTTACATTTTGTCCCTTAAGCGGAGCCCT | no hit |  |  |

Integrations numbers 1-8 were isolated from genomic DNA of fetuses, numbers 9-25 were from born offspring.
